# Circulating Tumor Cell Analysis: Technical and Statistical Considerations for Application to the Clinic

**DOI:** 10.1155/2010/426218

**Published:** 2009-12-13

**Authors:** Alison L. Allan, Michael Keeney

**Affiliations:** ^1^London Regional Cancer Program, London Health Sciences Centre, London, ON, Canada N6A 5W9; ^2^Lawson Health Research Institute, London, ON, Canada N6A 4V2; ^3^Department of Oncology, Schulich School of Medicine and Dentistry, University of Western Ontario, London, ON, Canada N6A 3K7; ^4^Department of Anatomy & Cell Biology, Schulich School of Medicine and Dentistry, University of Western Ontario, London, ON, Canada N6A 3K7; ^5^Special Hematology/Flow Cytometry, London Health Sciences Centre, London, ON, Canada N6A 5W9

## Abstract

Solid cancers are a leading cause of death worldwide, primarily due to the failure of effective clinical detection and treatment of metastatic disease in distant sites. There is growing evidence that the presence of circulating tumor cells (CTCs) in the blood of cancer patients may be an important indicator of the potential for metastatic disease and poor prognosis. Technological advances have now facilitated the enumeration and characterization of CTCs using methods such as PCR, flow cytometry, image-based immunologic approaches, immunomagnetic techniques, and microchip technology. However, the rare nature of these cells requires that very sensitive and robust detection/enumeration methods be developed and validated in order to implement CTC analysis for widespread use in the clinic. This review will focus on the important technical and statistical considerations that must be taken into account when designing and implementing CTC assays, as well as the subsequent interpretation of these results for the purposes of clinical decision making.

## 1. Introduction

Solid cancers are a leading cause of morbidity and mortality worldwide [1, 2], primarily due to the failure of effective clinical detection and treatment of metastatic disease in distant sites [3, 4]. The metastatic process is comprised of a series of sequential steps, and cancer cells must successfully complete each step in order to give rise to a metastatic tumor. These steps include dissemination of cancer cells from the primary tumor into the bloodstream (intravasation), survival in the circulation, arrest and extravasation into the secondary site, and initiation and maintenance of growth to form clinically detectable metastases [3–7]. Cancer cells may also disseminate from the primary tumor through the lymphatic system, although the lack of direct flow from the lymphatic system to other organs means that tumor cells escaping via this route must still enter the vascular system in order to be distributed to distant organs [3, 4, 8].

Given the multistep nature of the metastatic cascade, there should be several opportunities for early identification and therapeutic targeting of metastatic cells before they become a clinical problem. Indeed, in cancer patients with either metastatic or apparently localized disease, there is growing evidence that the presence of circulating tumor cells (CTCs) in the blood may be an important indicator of the potential for metastatic disease and poor prognosis (reviewed in [9–14]). Although CTCs have been recognized for over a century [15], a lack of sensitive technology precluded the detailed study of these cells until recently. However, technological advances have now facilitated the identification, enumeration, and characterization of CTCs using methods such as PCR [16–18], flow cytometry [19–21], image-based immunologic approaches [22–25], immunomagnetic techniques [26, 27], and microchip technology [28]. The ability to consistently enumerate, track, and characterize rare CTCs in cancer patients holds tremendous promise in terms of identifying the potential for metastatic disease at very early stages, managing risk stratification in the adjuvant setting, monitoring response to treatment, monitoring disease recurrence, and the prospective development of targeted therapies based on molecular characterization of CTCs [9–14]. However, the rare nature of these cells requires that very sensitive and robust detection/enumeration methods be developed and validated in order to implement CTC analysis for widespread use in the clinic. This review will therefore focus on the important technical and statistical considerations that must be taken into account when designing and implementing CTC assays, as well as the subsequent interpretation of these results for the purposes of clinical decision making.

## 2. Technical and Statistical Considerations for Optimal CTC Analysis

In metastatic cancer patients, it is estimated that CTCs in the peripheral blood can occur at a frequency of approximately 1 CTC per 10^5^–10^7^ peripheral blood mononuclear cells, and this frequency may be even lower (~1 in 10^8^) in patients with localized cancer [14, 29–31]. Accurate detection of rare events such as CTCs requires the ability to detect single cells with specific characteristics against a background of large numbers of other cells. In clinical applications of rare event detection, there is the added challenge of detecting the cells of interest in a limited sample volume and being able to accurately differentiate these cells from cell debris or other artifacts of sample preparation within a heterogeneous population of cells [29]. Furthermore, clinical tests need to be highly standardized and reproducible in order to be used for diagnostic, prognostic, and/or predictive purposes in patients. Assays designed to detect CTCs must therefore be carefully designed and validated with several important considerations in mind.

### 2.1. Specificity and Sensitivity

The rare nature of CTCs clearly requires an assay with a high degree of sensitivity in order to accurately detect cells down to a frequency of 1 in 10^7-8^. However, a second critical requirement centers around the specificity of the assay, from the point of view that a maximum number of true positive events need to be identified while at the same time minimizing false positive and false negative results. To identify CTCs in blood, investigators have taken the approach of exploiting phenotypic differences between epithelial tumor cells and cells of hematopoietic origin using markers specific to each population. For example, the highly conserved hematopoietic marker CD45 can be successfully employed as an exclusionary marker to identify leukocytes and eliminate them from subsequent analysis [32, 33]. However, the identification and utilization of a corresponding CTC-specific marker has proven to be more problematic, and in fact the inherent heterogeneity and genetic instability of tumors [34] make it unlikely that such a marker even exists. CTCs have instead been identified using two alternative approaches; those using tumor type-specific markers, and those using epithelial-specific markers.

Tumor type-specific markers offer the advantage of only identifying tumor cells that have disseminated from a particular tumor type. Markers of this type that have been used for CTC analysis include mammoglobin, HER2-neu, mucin 1 (breast cancer), prostate specific antigen (prostate cancer), carcinoembryonic antigen (colorectal and gastric cancer), and others [31, 35, 36]. Although these types of markers may offer the potential advantages of specifically identifying tumor cells rather than just epithelial cells and also providing some insight into their molecular characteristics, the major problem with tumor type-specific markers is that tumors are inherently heterogeneous, and there are currently no markers known that are expressed by every tumor cell within a given tumor type. Therefore, an underestimation of actual CTCs may occur.

In addition to tumor type-specific markers, a second approach that has been more widely used for detecting CTCs involves the use of epithelial-specific markers. These types of markers are theoretically expressed by all tumor cells of epithelial origin as well as by normal epithelial cells, although the latter are unlikely to be present in the peripheral bloodstream. The most commonly used markers in this category include epithelial cell adhesion molecule (EpCAM, also known as ESA, CD326, HEA125, or TACSTD1) and cytokeratins (CK) 7, 8, 18, 19, and/or 20 [11]. One potential problem with using epithelial-specific markers is that, although the majority of leukocytes do not express epithelial markers, they have occasionally been observed to become positive for such antigens when in an activated state [31, 37]. In addition, experimental studies have demonstrated that more aggressive and/or metastatic tumor cells often undergo a so-called epithelial-to-mesenchymal transition (EMT) which facilitates migration and invasion into secondary metastatic sites. Cells undergoing EMT show reduced cell-cell adhesion, altered morphology, gain of mesenchymal protein expression, and loss of epithelial marker expression [38–40]. This may include markers such as EpCAM and CKs that are used in CTC assays [41]. Therefore, similar to the issues with tumor type-specific markers, an underestimation of actual CTCs numbers (in particular the more metastatic cells) may occur. However, despite problems with both marker approaches, EpCAM and CK have emerged as the most widely accepted CTC markers because of their applicability across numerous different tumor types of epithelial origin [12]. The simultaneous use of multiple markers and continued research into novel CTC markers will hopefully lead to enhanced specificity and sensitivity of CTC assays in the future.

### 2.2. Sample Enrichment

In order to maximize sensitivity in rare event detection, most assays aiming to accurately detect events below a frequency of ~1 : 10^4^ generally apply a sample enrichment step to increase the likelihood of finding the events of interest. Enrichment approaches include either positive selection or negative selection using immunomagnetic methods, density gradient centrifugation, and/or cell size restrictive filtration [11, 32, 33, 42]. Positive selection involves the use of an antibody against the CTC target population (i.e., anti-EpCAM) attached to magnetic beads [26]. In contrast, negative selection uses an antibody against the nontarget or background population (i.e., anti-CD45 to identify leukocytes) [32, 33, 42]. Positive selection approaches usually result in fairly high purity of the sample recovered, since only CTCs will be selected. However, the recovery of CTCs may be lower than in negative selection approaches, since CTCs that have low or absent expression of EpCAM (or other positive selection markers) may be lost [41]. In addition, enrichment methods that employ a minimum number of processing steps are best in order to minimize CTC loss. Some CTC assays have been performed directly in whole blood without enrichment [23], although the stringency of this approach compared to others in the field remains controversial [43].

### 2.3. Statistical Considerations

Clinical blood tests are limited by the volume of blood that can be practically and ethically collected from patients. Since Poisson statistics apply when counting randomly distributed objects (CTCs) in a certain volume, an important consideration is whether the blood sample volume will be adequately large enough to accurately detect and enumerate a very small number of CTCs [29]. For cell-based assays such as flow cytometry, a simple calculation can be used to determine the size of the database/sample that will provide a given precision:

(1) $$r={\left(\frac{100}{\text{CV}}\right)}^{2},$$

where *r* is the number of events meeting the required criterion, and CV is the coefficient of variation of a known positive control (Table 1). In addition, in order to determine the statistical variation or range that can be expected around the “true” value, the standard deviation (SD) must also be taken into account; where SD equals the *√*target events counted, and the 95% confidence interval is equal to 2 × SD.

For example, if the desired CV is 10%, then: *r* = (100/10)^2^ = 100 events of interest. The true value would actually be somewhere between 80–120 events in 10^5^ total events, if the estimated frequency was 1 in 1000. Of note, this is close to the limit of detection of most routine clinical flow cytometry tests in the laboratory (i.e., detection of CD34^+^ cells in hematopoietic stem and progenitor cell transplants). Even at this level it has taken several years to standardize this process [44]. From the perspective of CTCs, if 6 CTCs were detected in a given sample with a CV as high as 40% (in the range of what has been shown to have prognostic significance in metastatic breast and prostate cancer patients [26, 45]), then the true value would actually be somewhere between 1 to 11 CTCs in 6.3 × 10^6^ leukocytes, if the estimated frequency was 1 in 10^7^. To reduce the CV to ~10% (and hence improve the accuracy of the assay), then many more (10^9^) events would need to assessed (Table 1). To put this into perspective with regards to a patient blood sample, 10 mL of blood would contain ~5 × 10^7^ leukocytes, assuming a leukocyte count in the low-normal range (5 × 10^9^/L), which is likely in a cancer patient on active treatment [46]. It is therefore not surprising that analysis of multiple blood samples from the same patient at the same time point significantly improves the probability of accurately detecting small numbers of CTCs [29]. These statistical considerations also suggest that some caution should be employed when using a threshold cutoff number (i.e., ≥5 CTC versus <5 CTCs) [26, 45] to stratify cancer patients into poor prognosis versus good prognosis groups for the purposes of clinical decision making.

Other factors also influence the statistical accuracy of a CTC assay, including assay efficiency with regards to the recovery/preservation of CTCs during sample preparation and/or enrichment (discussed earlier), as well as intra-operator/interlaboratory variability. The latter is particularly important when considering implementation of CTC assays for multicenter studies, especially for those CTC assays that may have a subjective component to the readout [29]. The success of rare event detection is therefore affected by many parameters, including quality of the starting sample, frequency of the events of interest, sample preparation, specificity and expression level of the chosen markers, robustness of the assay, and objective and reproducible readouts. All of these factors will contribute to the statistical probability of accurately detecting and quantifying rare events such as CTCs, and therefore are important to consider when designing and interpreting CTC assays for clinical use.

## 3. Currently Used Methods of CTC Analysis

Several methodological approaches have been used to detect rare CTCs, including PCR-based approaches [16–18, 47], flow cytometry [20, 21], image-based immunologic approaches [23–25], immunomagnetic techniques [26, 27], and microchip technology [28]. Each of these approaches has distinct advantages and disadvantages, with the most notable being sensitivity and specificity (Table 2). For example, PCR-based approaches have the advantage of being very sensitive, with a demonstrated lower detection limit in the clinical setting of approximately 1 tumor cell per 1,000,000 host cells (10^−6^) [16–18]. However, specificity can be a problem, since the amplification-based nature of PCR can result in false positives in the presence of even minimal sample contamination. In contrast, cytometric methods such as flow cytometry and laser scanning cytometry have high specificity due to their capacity for simultaneous analysis of multiple parameters on a cell-by-cell basis (i.e., DNA content, cell size, cell viability, expression of intra- and extracellular markers), but current systems are limited by their lower sensitivity (10^−4^-10^−5^) and thus by the blood volume that needs to be analyzed in order to accurately and reproducibly detect and enumerate very small numbers of CTCs [20, 21, 23, 48–50]. However, recent advances in cytometry technology will allow for more parameters to be assessed much more rapidly than previous generations of instruments. Indeed, current clinical flow cytometers being marketed can detect up to 10 fluorescence parameters at speeds on many thousand events/second. A recent paper by He et al. (2007) using a folate probe against ovarian cancer cells in twelve patients showed the ability to detect CTCs in the range of 10–150 CTCs/mL blood in nine patients (8 stage III, 1 stage IV), with the other three being stage I or II. Visual confirmation of the cells in the first 5 patients was carried out by staining with CA125. Studies such as this highlight the potential for exploitation of new surface receptors and new cytometry technology to identify CTCs [51].

The challenges of CTC analysis have led to the development of a number of innovative technologies specifically designed for this purpose. The CellSearch Circulating Tumor Cell Test (Veridex) is the only CTC test that is currently approved by the U.S. Food and Drug Administration for CTC analysis in metastatic breast, prostate, and colorectal cancers [26, 52, 53]. The CellSearch system consists of two components: the CellTracks AutoPrep System and the CellTracks Analyzer. The Autoprep System carries out automated and standardized immunomagnetic cell enrichment using antibodies targeting epithelial cell adhesion molecule (EpCAM), and subsequent labeling with fluorescent antibodies specific for epithelial cells (CK 8, 18, and 19) and leukocytes (CD45). The CellTracks Analyzer is comprised of a semi-automated fluorescence microscope and analysis software, and distinguishes epithelial tumor cells from leukocytes based on positive staining for CK, negative staining for CD45, cell size, cell morphology, and positive staining with the DNA stain 4′, 6-diamino-2-phenylindole (DAPI) [26]. 

Additional unique and promising systems for CTC analysis have also recently been reported. For example, the “CTC-chip” is a silicon microchip containing thousands of microposts coated with anti-EpCAM. Microfluidics is used to pneumatically push whole blood over the surface of the CTC-chip, and EpCAM-positive CTCs are captured and confirmed as CTCs via fluorescence microscopy analysis of CK expression [28]. Other investigators have shown that CTCs can be identified using microfiltering combined with electrolysis and RT-PCR [47], or by secretion of epithelial-specific or tumor type-specific soluble proteins using the EPISPOT technology [54]. Although all of these new approaches show remarkable sensitivity and the potential recovery of cells for further molecular characterization, their development is somewhat less mature than that of the CellSearch with regards to standardization and clinical utility.

In summary, several methods are available to detect CTCs, all of which have distinct advantages and disadvantages (Table 2). Numerous clinical studies using these different CTC assays have been conducted across many different tumor types and different disease stages. However, it remains difficult to draw any definitive conclusions regarding the value of CTC enumeration for clinical practice, based not only on the variable assays used but also on the fact that many studies have a small number of patients and controls and are not conducted in multicenter settings [14, 31]. Nevertheless, results obtained from these studies do suggest a promising association between CTCs and clinical oncology parameters, and this is discussed in the next section.

## 4. Application of CTC Analysis as a Tool for Clinical Decision Making

### 4.1. Detection and Enumeration of CTCs in Patients with Early-Stage Disease

New prognosis tools in the setting of early-stage cancer have the potential to improve patient quality of life and enhance clinical decision making. Currently, the use of well-established prognostic indicators (such tumor size or grade) to predict outcome is helpful but imperfect, owing mainly to tumor plasticity and the reliance on subjective assessment criteria. Similarly, although some specific molecules are currently in clinical use as prognostic markers of patient outcome (i.e., HER-2 for breast cancer), these too are imperfect as a result of tumor heterogeneity. This uncertainty in predicting disease outcome often results in undertreatment or overtreatment: some patients who need systemic therapy to treat undetected metastatic disease may be missed, and other patients who have been successfully treated by local surgery and radiation and do not require systemic therapy may be unnecessarily exposed to toxic side-effects. There is therefore a clear need for improved tools that could be used to accurately and reliably predict disease outcome.

A few studies have demonstrated that CTCs can be observed in ~20%–40% of patients with early-stage breast cancer using PCR-based assays for CK-19 [18, 55–58] and in ~10% of early-stage patients using the CellSearch system [35]. In many cases this can be correlated with poorer outcome with regards to both progression-free and overall survival, regardless of nodal status or adjuvant therapy [18, 35, 55–58]. Similarly, in patients undergoing curative intent resection surgery for early-stage colorectal cancer, CTCs identified by both CK-20 and CEA mRNA positivity within 24 hours of resection were indicative of relapse, particularly when combined with nodal status [59]. However, studies in localized prostate cancer did not show such a relationship, where analysis of CTCs by either the CellSearch system or RT-PCR for various transcripts (PSA, KLK2 [kallikrein-related peptidase 2], and PSCA [prostate stem cell antigen]) demonstrated that CTCs were rarely observed in patients with localized prostate cancer [60].

At present, not enough evidence is available regarding how CTC detection and enumeration might be useful for making clinical decisions in the early-stage/adjuvant setting [35]. Although the greatest amount of data is available for breast cancer, the American Society of Clinical Oncology (ASCO) Tumor Marker Guidelines Panel has still recommended that currently available CTC results in the early breast cancer setting must be considered level III at best evidence (where level I is best evidence and level IV is worst evidence), and therefore insufficient to apply to standard practice at present [61]. 

### 4.2. Detection and Enumeration of CTCs in Patients with Metastatic Disease

Despite significant improvements in detection and treatment of early-stage cancer, the majority of patients with metastatic cancer will ultimately die of their disease [1]. In the metastatic setting, the clinical goal is therefore to choose the therapy regimen that will have the highest likelihood of response and/or palliation, as well as the lowest risk of toxicity. This therapy is generally continued until either excessive toxicity occurs or evidence of disease progression indicates that the therapy is no longer effective [35]. Therefore, in the metastatic setting, new clinical tools that can accurately track disease progression and/or predict response to therapy would be particularly useful.

The majority of evidences supporting the use of CTCs as clinical decision making tools in patients with metastatic cancer have been obtained using the CellSearch system and analysis of 7.5 mL blood samples. The first of these major studies was carried out in breast cancer patients, where Cristofanilli et al. (2004) observed that ~60%–70% of metastatic breast cancer patients have ≥2 CTCs, whereas CTCs were very rarely observed in normal control subjects [26, 62]. Statistically, it has been shown that patients with ≥5 CTCs at baseline had poorer progression-free and overall survival than patients with <5 CTCs [26]. Subsequent studies demonstrated similar results for metastatic prostate and colorectal cancers, with the identified threshold number needed for stratification into the poor prognosis group being ≥5 CTCs at baseline for prostate cancer patients, and ≥3 CTCs at baseline for colorectal patients [45, 52, 53]. These studies also showed promising results with regards to CTCs serving as possible surrogate markers for early treatment response. In metastatic breast, prostate, and colorectal cancer patients, a decrease in CTC levels 2–5 weeks after starting systemic therapy was correlated to improved progression-free and overall survival [26, 45, 52, 53, 63]. In some cases, CTC analysis was found to be better for predicting treatment response than commonly employed methods such as radiologic assessment (in breast cancer) [64] and measurement of PSA (in prostate cancer) [45].

The observation that CTC levels during treatment may serve as a marker for treatment efficacy is interesting, but further studies are needed to validate this as a routine clinical tool. The Southwest Oncology Group (SWOG S0500) trial is designed to test the strategy of changing therapy versus maintaining therapy for metastatic breast cancer patients who have elevated circulating tumor cell (CTC) levels at first follow-up as assessed by the CellSearch system in 7.5 mL blood samples [65]. Opened in October 2006, this is a multicenter partially randomized trial that aims to enroll 500 patients with confirmed stage IV (metastatic) disease undergoing first-line chemotherapy. Patients with <5 CTCs at baseline (low risk) will receive standard chemotherapy without change. Patients with ≥5 CTCs at baseline (high risk) will undergo a second blood draw after completion of the first course of chemotherapy. Of these patients, women with <5 CTCs after completing one course of chemotherapy will continue to receive the same chemotherapy regimen with no change. Patients with ≥5 CTCs after completion of one course of chemotherapy will be randomized to either continue with the same chemotherapy or switch to a different regimen. This trial will hopefully provide information regarding whether it is more effective to change treatment regimens at the time of CTC increase or wait until disease progression, thus reflecting the efficacy (or lack of efficacy) of chemotherapy in individual patients and facilitating better treatment decisions.

Similar to the early-stage setting discussed above, current ASCO guidelines do not yet support the use of CTC assays for clinical management decisions in metastatic breast cancer (or other cancers), mainly due to the wide range of methodologies being used and the need for further clinical validation of such tests [57]. However, the uniqueness of the metastatic setting as often being an end-stage/palliative situation has led some clinicians to start incorporating CTCs into their decision making paradigm, in particular for those patients in which standard clinical, serologic, and/or radiographic findings are noninformative [35].

### 4.3. Statistical Considerations for Application of CTC Enumeration to Clinical Practice

Although the growing clinical data supporting the use of CTC detection and enumeration in clinical oncology is promising, the technical and statistical limitations discussed earlier must be taken into consideration before widespread application of such assays into clinical practice. When the first prospective multicenter study of CTCs using the CellSearch system was published in 2004, the critical role of 5 CTCs per 7.5 mL of blood in determining poor prognosis versus good prognosis in breast cancer patients was puzzling, given the inherently rare nature of these cells and the variability associated with rare event detection [29]. In fact, this number is more likely to be a somewhat arbitrary number with more basis in statistical significance between prognosis groups than actual clinical or biological relevance. From a biological perspective, it is logical to hypothesize that the greater the number of CTCs present in a patient's blood, the more aggressive the disease and the poorer the outcome will be. However, reanalysis of the data from Cristofanilli et al. (2004) demonstrated that median survival does not decrease further when greater than 5 CTCs (i.e., 6–100 CTCs) versus 5 CTCs are detected [26, 29]. This was somewhat surprising and suggests that CTCs need to be present at a certain concentration in order to be detected at any level. Statistically there may be a higher probability of correctly identifying CTCs when >5 are detected, and/or there may be a greater chance of incorrect identification when 1–4 CTCs are detected. This reanalysis concluded that likely the presence of even 1 CTC in 7.5 mL of blood has clinical relevance with regards to disease outcome [29]. Combined with the issues of statistical variability in rare event detection discussed earlier, these results suggest that caution should be employed when using a threshold number (i.e., ≥5 CTC versus <5 CTCs) [26, 45] to stratify cancer patients into poor prognosis versus good prognosis groups for the purposes of clinical decision making. A more appropriate application of CTC analysis may be for predictive purposes in terms of determining a patient's response to therapy (i.e., by tracking significant changes in CTC numbers within individual patients over time in response to treatment), and studies such as the SWOG S0500 trial may provide support for this. In addition, a growing number of studies are moving beyond enumeration and towards molecular characterization of CTCs. This type of analysis has less statistical problems associated with it than those enumeration, and could provide extremely valuable information regarding the identification of specific targets for therapy.

### 4.4. Future Perspectives-Molecular Characterization CTCs

The possibility of assessing the molecular characteristics of CTCs holds tremendous potential both from research and clinical standpoints. Currently, assessment of the primary tumor for specific molecular characteristics (i.e., estrogen receptor [ER] and HER-2 status in breast cancer) is carried out on primary tumor tissue. However, this assessment is rarely repeated on metastatic tissues, largely due to the challenges of finding and obtaining biopsies of metastatic lesions [66]. The clinical implications of this are that treatment decisions regarding targeted therapies such as hormonal therapy or Herceptin (in the case of HER-2) are made based on the features of the primary tumor. However, studies have demonstrated that tumor cells “evolve” during progression from primary to metastatic disease often displaying very different molecular characteristics in a metastatic site than in the primary tumor [67–70]. Since CTCs are hypothesized to be the intermediaries between primary and metastatic disease and/or surrogates of a patient's metastatic tumor [33, 71], molecular characterization of CTCs may provide an opportunity for noninvasive “real-time” biopsies during disease progression in order to track these molecular changes and potentially incorporate them into clinical decision making.

**Figure 1:**
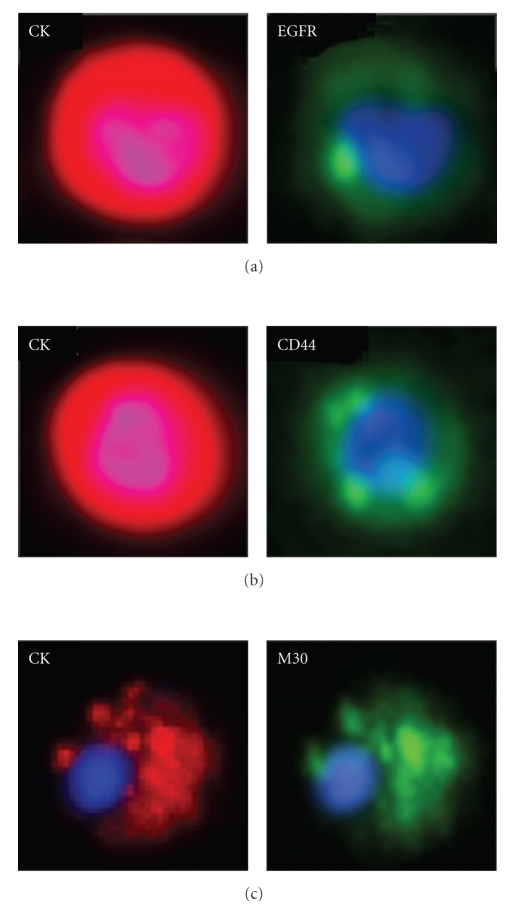
Specific molecular features of CTCs can be analyzed via secondary phenotyping using the CellSearch system (Veridex) (a)–(c). Breast cancer CTCs in 7.5 mL of blood were processed on the CellTracks AutoPrep system using the CellSearch CTC kit and additional characterization antibodies against either (a) EGFR (Veridex), (b) CD44 (4 *μ*g/mL; BD BioSciences), or (c) M30 (3.7 *μ*g/mL; Alexis Biochemicals). Samples were then analyzed by the CellTracks Analyzer II. CTCs were identified and enumerated via positive staining for CK and DAPI (*respective red and blue staining in left panels*), negative staining for CD45 (*not shown*), and size and morphological characteristics. The additional FITC channel (*green staining in right panels*) was exploited for identification of molecular characteristics in individual CTCs. Expression of markers such as (a) EGFR and (b) the cancer stem cell marker CD44 may provide information regarding disease aggressiveness and/or indicate patient suitability for specific targeted therapies. Apoptosis markers such as (c) M30 (caspase-cleaved CK18) and the corresponding morphological characteristic of apoptotic membrane blebbing could provide information in patients on active treatment regarding efficacy of therapy and antitumor effects. The sample shown in (c) was exposed to palitaxel chemotherapy prior to CTC analysis.

A number of studies have reported characterization of specific molecular features of CTCs, either directly through RT-PCR or through secondary phenotyping and genotyping of CTCs after immunomagnetic selection and enumeration (Figure 1). Examples of such molecular characteristics include EGFR (expression and/or mutation), uPAR, HER2, IGF1R, and markers for apoptosis such as M30 (useful for assessing therapy response) [66, 72–76]. Some studies have also recovered sufficient numbers of CTCs to carry out gene expression profiling [77]. Reports have also shown a value for fluorescence in situ hybridization (FISH) analysis in CTCs, which allows the assessment of cytogenetic changes (such as gene translocation or amplification) in a patient's disease during evolution towards a metastatic phenotype [66, 76, 78, 79]. For example, Meng et al. (2004) used FISH to demonstrate that ~38% of metastatic breast cancer patients who were initially HER2-negative (based on their primary tumor) acquired amplification of HER-2 in their CTCs. When treated with Herceptin based on CTC HER-2 amplification, some patients demonstrated a partial or complete response [66].

Although large-scale clinical data is still lacking with regards to how molecular characterization of CTCs could be used as a clinical decision making tool, this type of analysis holds tremendous promise with regards to gaining a better biological understanding of the metastatic process, improved stratification of patients, and/or the prospective development of tailored, targeted therapies.

## 5. Summary and Conclusions

In summary, technology development and interest in the area of CTC analysis is advancing rapidly. CTCs have great potential as surrogate markers for cancer progression and treatment, particularly in the metastatic setting. However, we believe that many technical and statistical issues remain to be resolved before CTC analysis can be considered for widespread application to the clinic. The success of CTC detection and enumeration is influenced by many parameters including quality of the starting sample, frequency of CTCs, sample preparation, specificity and expression level of the chosen markers, robustness of the assay, and objective and reproducible readouts, including intrareader and interlaboratory variability. All of these factors will contribute to the statistical probability of accurately detecting and quantifying rare events such as CTCs, and therefore are important to consider when designing and interpreting CTC assays for clinical use.

## Figures and Tables

**Table 1:** Determination of database/sample size that will provide a given precision in rare event analysis.^(a)^

Desired CV (%) →		1	5	10	20	40
*r* = no. of events of interest →		10000	400	100	25	6
When occurring at a frequency of		Total no. of events which must be collected^(b)^				
(%)	1 : *n*					

10	10	10^5^	4 × 10^3^	10^3^	2.5 × 10^2^	6.3 × 10^1^
1	100	10^6^	4 × 10^4^	10^4^	2.5 × 10^3^	6.3 × 10^2^
0.1	1000	10^7^	4 × 10^5^	10^5^	2.5 × 10^4^	6.3 × 10^3^
0.01	10,000	10^8^	4 × 10^6^	10^6^	2.5 × 10^5^	6.3 × 10^4^
0.001	100,000	10^9^	4 × 10^7^	10^7^	2.5 × 10^6^	6.3 × 10^5^
0.00001^(c)^	10,000,000	10^11^	4 × 10^9^	10^9^	2.5 × 10^8^	6.3 × 10^7^

^(a)^For cell-based assays such as flow cytometry, a simple calculation can be used to determine the size of the database/sample that will provide a given precision: *r* = (100/CV)^2^; where *r* is the number of events meeting the required criterion, and CV is the coefficient of variation of a known positive control. Modified from http://www.icms.qmul.ac.uk/flowcytometry/uses/rareeventanalysis/index.html, Queen Mary, University of London.

^(b)^With a WBC count in the low-normal range (~5 × 10^9^/L), 10 mL of blood would contain ~5 × 10^7^ events.

^(c)^Estimated frequency of CTCs in the peripheral blood of cancer patients.

**Table 2:** Advantages and disadvantages of currently used methods of CTC analysis.

Method	Estimated sensitivity	Advantages	Disadvantages	Selected references
PCR-based approaches	10^−4^–10^−6^	(i) Rapid, quantitative (ii) High sensitivity (iii) Small sample volume required	(i) Does not allow for cell-by-cell analysis (ii) Does not discriminate between viable and nonviable cells (iii) Low specificity (iv) Technical issues with mRNA degradation, etc.	[16–18, 47, 55–58]

Flow cytometry	10^−4^-10^−5^	(i) Rapid, quantitative (ii) Cell-by-cell analysis (iii) Multiparameter (iv) High specificity (v) Identification of viable versus nonviable cells (vi) Potential to sort CTCs for additional characterization	(i) Limited sensitivity (ii) Requirement for large sample volume unless sample enrichment used (iii) No visual confirmation of cell specificity (iv) Technically and analytically challenging	[20, 21]

Laser scanning cytometry	10^−4^-10^−5^	(i) Rapid, quantitative (ii) Cell-by-cell analysis (iii) Multiparameter (iv) High specificity (v) Identification of viable versus nonviable cells (vi) Morphological analysis	(i) Limited sensitivity (ii) Technically and analytically challenging	[23–25]

CellSearch (Veridex)	10^−7^	(i) High sensitivity and specificity (ii) Automated, quantitative (iii) Highly reproducible (iv) Moderate sample volume needed (v) Identification of viable versus nonviable cells (vi) Commercially available (vii) Only assay with FDA approval	(i) Limited analysis parameters (ii) Use of EpCam to capture CTCs may miss some tumor cells (iii) Multiple enrichment and processing steps may result in loss of CTCs (iv) Partially subjective readout	[26, 45, 52, 53]

CTC microchip	10^−7^+	(i) High sensitivity and specificity (ii) Quantitative (iii) Minimal processing and shear stress (iv) Identification of viable versus nonviable cells (v) Potential to recover CTCs for additional characterization	(i) Technology is not commercially available (ii) Use of EpCam to capture CTCs may miss some tumor cells (iii) Partially subjective readout	[28]

EPISPOT	10^−7^+	(i) High sensitivity and specificity (ii) Quantitative (iii) Multiparameter (iv) Only viable tumor cells are detected	(i) Requires 48-hour culture of isolated CTCs before analysis	[54]
